# Rotational vs. Straight Landings: Exploring Task-Specific Responses to Inform ACL-Injury Risk Screening

**DOI:** 10.5114/jhk/200765

**Published:** 2025-09-23

**Authors:** Parunchaya Jamkrajang, Sarit Suwanmana, Chuanpis Boonkerd, Jasper Verheul

**Affiliations:** 1 College of Sports Science and Technology, Mahidol University, Nakhon Pathom, Thailand.; 2 Department of Physical Therapy, Faculty of Allied Health Sciences, Thammasat University, Pathum Thani, Thailand.; 3 Cardiff School of Sport and Health Sciences, Cardiff Metropolitan University, Cardiff, UK.

**Keywords:** functional task, anterior cruciate ligament, injury screening, sex differences

## Abstract

Rotational landing tasks have the potential to support screening methods for anterior cruciate ligament (ACL) specific injury risk. However, alterations in lower-limb kinematics and kinetics during rotational landings, and sex-specific responses, are currently largely unexplored. This study, therefore, explored the differences in lower-limb kinematic and kinetic characteristics between rotational and straight landings, and the sex-specific responses to rotational landings. Thirty-six healthy team-sport athletes (eighteen males and eighteen females) performed straight bilateral and unilateral landings, and rotational (clockwise and counterclockwise) landings, from a box while lower-limb kinematics and ground reaction forces (GRFs) were recorded. Rotational landings were found to emphasise (p < 0.001) hip flexion angles at initial contact and peak vertical GRF. Differences between males and females (p < 0.001) were identified during rotational landings (but not straight landings) for peak ankle dorsiflexion and time to peak vertical GRF, with significant task-sex interactions. Compared to the bilateral landing, unilateral tasks affected the magnitude or highlighted sex-specific differences for nine and one biomechanical characteristics, respectively. Together, these outcomes provide further insights into lower-limb kinematic and kinetic responses to rotational landings. These findings offer additional support for the use of rotational, as well as unilateral elements, for ACL-injury risk screening practice.

## Introduction

Functional tasks, such as vertical landings and jumps, are widely used to screen for athletes at risk of common injuries. The aim of such screening approaches is to identify injury-related adjustments in biomechanical movement strategies. For example, altered lower-limb kinematics and kinetics during landing and jumping tasks, have been observed both prior to (Goerger et al., 2015) and after (Jones et al., 2025) occurrence of the anterior cruciate ligament (ACL) rupture, i.e., an injury with a high burden (incidence x severity) across various team-sports populations (Moses et al., 2012). Biomechanical screening of kinematic and kinetic variables during landing tasks has thus been widely employed to identify athletes at risk of ACL injury (either primary or secondary) (Hewett et al., 2005; Leppänen et al., 2017; Pedley et al., 2020). Nevertheless, current ACL-injury screening methods have been criticised for a lack of task sensitivity or specificity (Bahr, 2016; Krosshaug et al., 2016). Careful consideration of the aptness of landing tasks used for injury-screening purposes is thus essential.

Large ACL strains and the consequent rupture are primarily caused by a combination of forces and moments (e.g., knee abduction moments in combination with anterior shear force) in multiple directions (Bates et al., 2019; Markolf et al., 1995; Koga et al., 2010; Quatman et al., 2010). Given these multiplanar injury mechanisms of the ACL rupture, it is plausible that screening tasks should likewise be sufficiently inclusive of multiplanar movement, to highlight biomechanical deficiencies in at-risk individuals. Nevertheless, straight landing tasks (i.e., movement in the sagittal plane only) are still most commonly used in ACL-injury screening practice. Recent research has shown that the angle and the direction of rotational landings can substantially affect ACL-injury related knee joint angles and moments in the sagittal and frontal plane (Kunugi et al., 2020; Sinsurin et al., 2013, 2017). In addition, biomechanical characteristics displayed during rotational landings have been shown to be sufficiently sensitive to differentiate between various types of athletes (Taylor et al., 2017). Accordingly, it has been suggested that rotational landing tasks are better suited to identify individuals at ACL-specific injury risk, compared to straight landings (Fox et al., 2016; Schweizer et al., 2022). However, exactly which kinematic or kinetic variables are emphasised during rotational landings is yet largely unexplored and requires further examination.

Female athletes are well documented to sustain a higher rate of ACL injuries compared to males (Agel et al., 2005; Arendt and Dick, 1995; Waldén et al., 2011). Accordingly (and not surprisingly), landing biomechanics during common ACL-injury screening tasks are sex dependent (Beaulieu and McLean, 2012; Fagenbaum and Darling, 2003; Lephart et al., 2002). Between-sex differences in landing mechanics can, however, vary across different types of landings (Butler et al., 2013). Especially for key kinematic and kinetic ACL-injury risk factors, it is thus desirable if differences between male and female athletes can be appropriately highlighted by functional tasks used for injury screening. This ability to measure and monitor distinct between-sex differences will enable adequate sex-specific (rather than generic) monitoring of ACL-injury risk, making the specificity of landing tasks an important consideration for injury-risk screening purposes. However, although it is likely that males and females respond differently to rotational landings, it remains to be determined if rotational landings can highlight sex-specific differences well.

The effects of the rotational landing on lower-limb kinematics and kinetics, and sex-specific responses, are currently largely unexplored. The aims of this study were, therefore, twofold: first, to explore the differences in lower-limb kinematic and kinetic characteristics used for ACL-injury risk screening between rotational and straight landings, and second, to examine sex-specific responses to rotational landings compared to straight landings. It was hypothesised that rotational landings would emphasise kinematic and kinetic factors typically examined for screening purposes and could highlight between-sex differences in lower-limb biomechanics more adequately than straight landing tasks.

## Methods

### Participants

A cohort of 36 healthy athletes (eighteen males: age 23 ± 2 yrs, body mass 72 ± 14 kg, body height 173 ± 6 cm; eighteen females: age 23 ± 3 yrs, body mass 56 ± 9 kg, body height 162 ± 5 cm) volunteered for this study. Participants were required to actively participate in team sports (e.g., soccer, basketball, volleyball) for at least three sessions per week (≥1 hour per session) and were free from musculoskeletal lower-limb injuries over a twelve-month period before participating. Participants were fully informed about the study’s purpose, procedures, and potential risks before providing their written informed consent to participate. The number of required participants was determined using an a priori power analysis in G*Power (v.3.1.9.7), which indicated that a sample size of 36 participants was sufficient to reliably detect a large effect size (ηp^2^) of 0.14 (Cohen, 2013) with 95% power at a significance level (ɑ) of 0.003125 (i.e., 0.05/16; see below). All procedures complied with the 1964 Declaration of Helsinki and its later amendments and were approved by the Thammasat University Human Research Ethics Committee, Pathum Thani, Thailand (approval code: 073/2565; approval date: 25 August 2022).

### Design and Procedures

**Figure 1:**
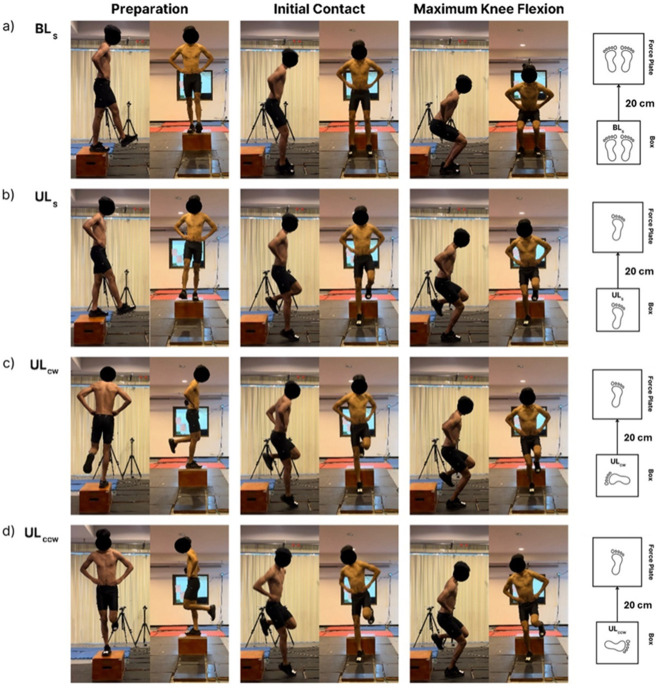
The landing tasks performed were a) a straight bilateral landing (BL_S_), b) a straight unilateral landing (UL_S_), c) a clockwise unilateral landing (UL_CW_), and d) a counterclockwise unilateral landing (UL_CCW_). Preparation for the movement, initial contact with the ground, and the moment of maximum knee flexion, are shown in the first, second, and third columns, respectively. The fourth column shows the feet in contact with the box and the force plate during and throughout each of the four landing tasks.

Upon arrival at the biomechanics laboratory, participants engaged in a warm-up routine consisting of lower-limb dynamic stretching exercises for ten minutes. After this, they completed three trials of four landing tasks: straight bilateral and unilateral landings (BLs, ULs), and rotational unilateral landings in the clockwise (UL_CW_) and counterclockwise (UL_CCW_) directions. All participants were fully instructed in the required landing tasks and then allowed to practice and familiarise themselves with the movements. Landings were performed in randomised counterbalanced order. Participants performed BLs and ULs by standing on a jumping box (height: 0.3 m) with both hands on the iliac crest and dropping onto a ground-embedded force plate (0.6 x 0.4 m, Kistler Instrument AG, Winterthur, Switzerland) with both or one leg, respectively. Rotational unilateral landings were performed in clockwise (UL_CW_) and counterclockwise (UL_CCW_) directions. Rotational landings required participants to stand on the dominant leg with the knee extended and hands on the iliac crest, and perform a rotational landing from a jumping box, maintaining minimal knee bending, and landing on the center of the force plate with their dominant leg. The distance between the jumping box and the center of the force plate was 0.2 m. The preferred leg for ball kicks was referred to as the dominant leg. All four landing tasks are visually represented in Figure 1. During the landings, participants had their eyes open, they were instructed to look forward and balance as quickly as possible and hold the single-limb stance for five seconds. Between each trial, participants rested for 30 s, and a five-minute rest interval was allowed between landing tasks. A trial was stopped and repeated if a participant's opposite foot touched the floor, if the foot moved after the landing or if the hands were used to regain balance.

Sixteen retroreflective markers were attached to each participant’s body, following the lower-limb Plug-in-Gait marker set (Vicon, Oxford, UK). During the landing tasks, three-dimensional marker positions and ground reaction force (GRF) were recorded using nine motion capture cameras (BTS DX 5000, BTS Bioengineering, Milan, Italy) and a force plate (0.6 x 0.4 m, Kistler Instrument AG, Winterthur, Switzerland) at a sampling frequency of 200 and 1000 Hz, respectively. Smart tracker and Smart analyser software (v.1.10.445.0, BTS bioengineering, Milan, Italy) were used to track, digitise, and interpolate any missing marker-trajectory data. Marker trajectories and GRF data were then exported to Visual 3D (v.2023.05.1, Has Motion Inc., Kingston, Ontario, Canada) for further processing. The three-dimensional marker trajectories and GRF were filtered using a 20-Hz lowpass Butterworth filter, after which inverse dynamics were used to estimate lower-limb joint moments.

Initial contact (IC) of each landing trial was determined as the moment when the vertical ground reaction force exceeded 10 N. Several common kinematic and kinetic variables that have been used to assess ACL injury risk during landings were calculated (Bathe et al., 2023; Peterson and Green, 2023; Sadeqi et al., 2023). Kinematic variables included the hip, knee, and ankle joint angles, and knee angular velocity, in the sagittal and frontal planes at the moment of IC. Kinetic variables included the peak hip, knee, and ankle joint moments in the sagittal and frontal planes, and peak vertical GRF and time to peak vertical GRF (from IC). Peak vertical GRFs and joint moments were normalised to each participant’s body weight and body mass, respectively. In addition, knee joint stiffness was estimated as the ratio of changes in the joint moment and flexion between IC and the peak joint flexion moment during the landing (Maloney et al., 2018; Serpell et al., 2012) according to the following formula:


$$K_{knee}\left(\frac{\text{Nm}}{\deg}\right)=\frac{\Delta M_{knee}}{\Delta \theta_{knee}}$$

### Statistical Analysis

Statistical analyses were performed using IBM SPSS Statistics (v.30, SPSS Inc, Chicago, USA). Mean and standard deviation values for each kinematic and kinetic variable were determined across all trials and participants. The data distribution was assessed using Shapiro-Wilk tests. A mixed model ANOVA was used to assess the effect of sex (male vs. female) and task (BLs vs. ULs vs. UL_CW_ vs. UL_CCW_). Post-hoc tests were conducted to identify the locations of significant effects. After Bonferroni correction, the level of statistical significance was set at α = 0.003125 (i.e., 0.05/16). For better comparability across studies (Knudson, 2009), effect sizes were determined by calculating partial eta squared (ηp^2^). Effect sizes were categorized as small (ηp^2^ = 0.01), medium (ηp^2^ = 0.06) or large (ηp^2^ = 0.14) effects (Cohen, 2013).

## Results

**Figure 2:**
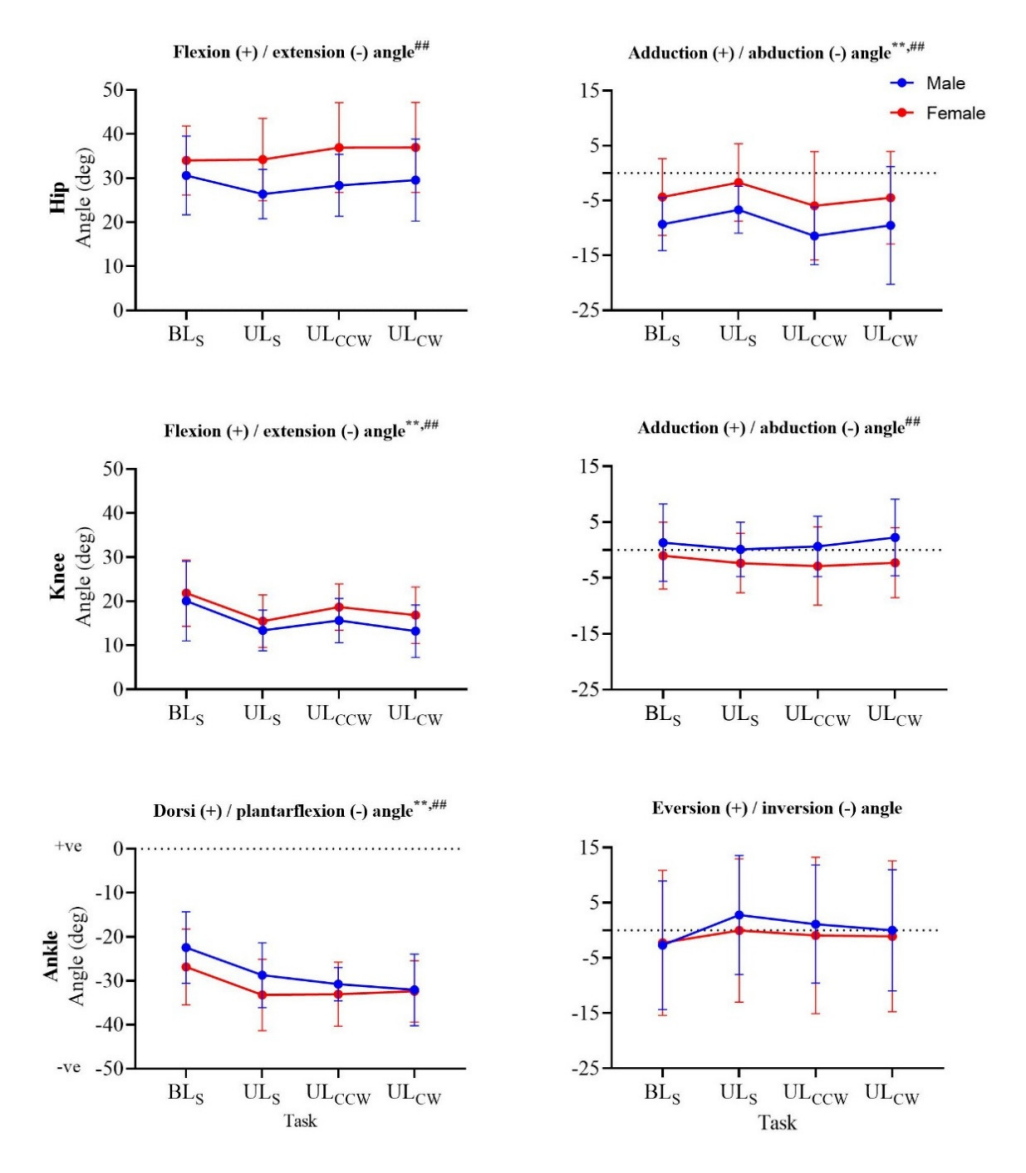
Joint angles at initial contact in the sagittal (left column) and frontal (right column) planes of all four landing tasks for males (blue) and females (red). ^#^ significant main effect (p < 0.05) of sex; ^##^ significant main effect (p < 0.01) of sex; ^**^ significant main effect (p < 0.01) of the task

A total of 432 successful landings were included in the analysis (108 BLs; 108 ULs; 108 UL_CW_; 108 UL_CCW_). The hip was significantly more flexed (*p* < 0.001, ηp^2^ = 0.18) and less adducted (*p <* 0.001, ηp^2^ = 0.13) at IC for females compared to males (Figure 2). Hip flexion was significantly increased in females for all unilateral landing tasks (*p <* 0.001), whereas hip adduction was significantly decreased in females for BLs, ULs, and UL_CCW_ (*p <* 0.001). There was also a significant main effect of the task on hip adduction (*p <* 0.001, ηp^2^ = 0.18) and flexion (*p <* 0.001, ηp^2^ = 0.06). In addition, there was a significant interaction between sex and task for the hip flexion angle at IC (*p =* 0.003, ηp^2^ = 0.05), with between-sex difference being larger for unilateral compared to the bilateral landing tasks.

Although there was no main effect of sex on knee flexion or adduction at IC (Figure 2), during the rotational landings, females landed with a significantly more flexed (UL_CW_, UL_CCW_, *p =* 0.003) and less adducted (UL_CCW_, *p <* 0.001) knee compared to males. Significant main effects for the landing task were found for knee flexion (*p <* 0.001, ηp^2^ = 0.29) and adduction (*p <* 0.001, ηp^2^ = 0.07) at IC.

The ankle plantarflexion (*p <* 0.001, ηp^2^ = 0.33) and eversion (*p <* 0.001, ηp^2^ = 0.16) angles at IC were both significantly affected by changes in the landing task (Figure 2), but not sex. There were significant increases in ankle plantarflexion between BLs and the unilateral landing tasks (*p <* 0.001).

**Figure 3:**
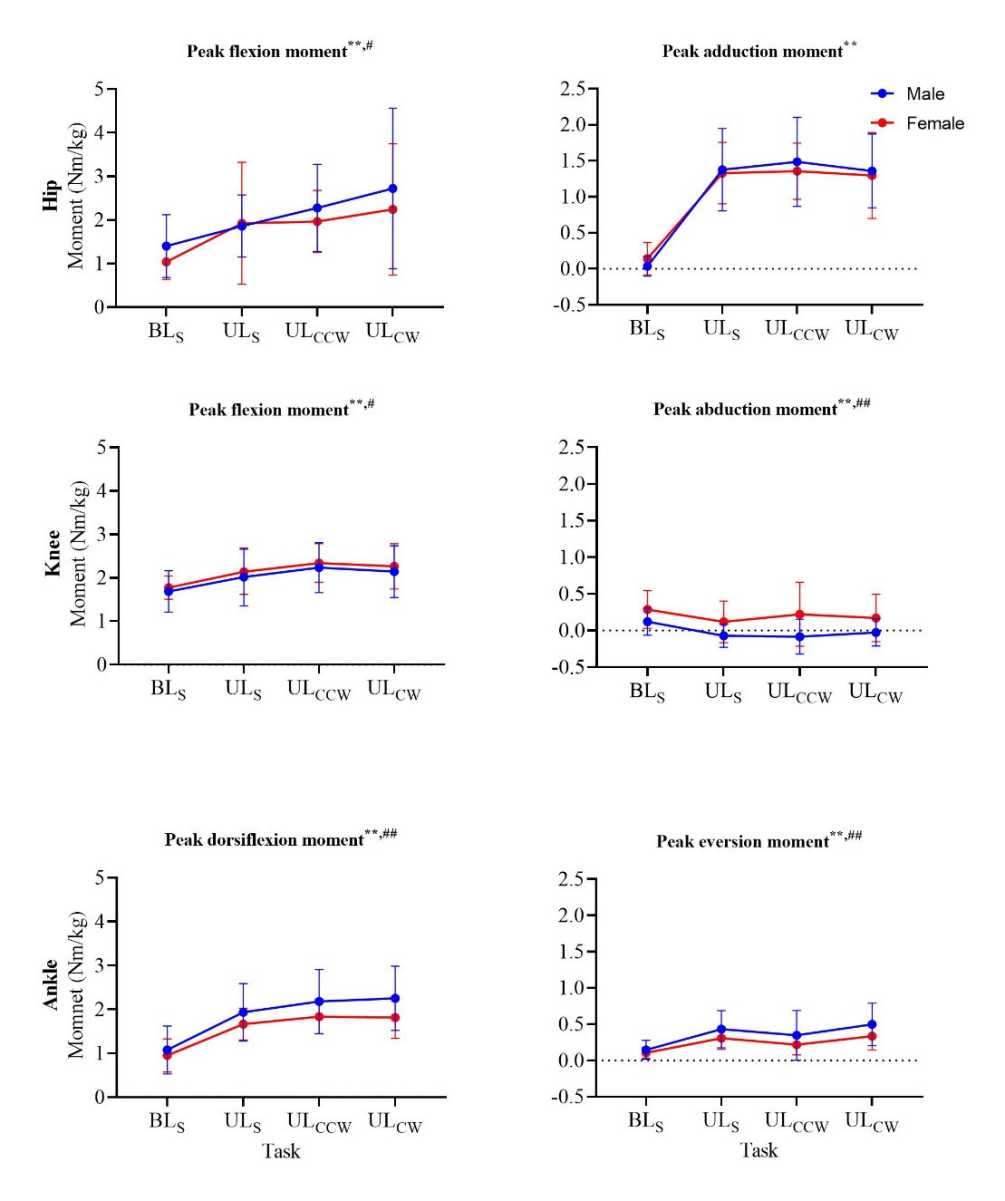
Peak joint moments in the sagittal (left column) and the frontal (right column) plane of all four landing tasks for males (blue) and females (red). ^#^ significant main effect (p < 0.05) of sex; ^##^ significant main effect (p < 0.01) of sex; ^**^ significant main effect (p < 0.01) of task (p < 0.01)

Peak hip flexion moments were significantly affected by the landing task (*p <* 0.001, ηp^2^ = 0.25), but not sex (Figure 3). Hip flexion moments were higher for all unilateral landings compared to bilateral landing (*p <* 0.001). The peak hip adduction moment was significantly affected by the landing tasks (*p <* 0.001, ηp^2^ = 0.74) and increased from bilateral to unilateral landings, but did not differ between unilateral tasks or males and females.

The type of the landing task significantly affected the peak knee flexion moments (*p <* 0.001, ηp^2^ = 0.30) (Figure 3), with significant differences between BLs and all unilateral landings (*p <* 0.001), and ULs and UL_CCW_ (*p <* 0.001). Peak knee abduction moments were significantly higher for females compared to males across tasks (*p <* 0.001, ηp^2^ = 0.16). Moreover, a significant main effect for the landing task was found (*p <* 0.001, ηp^2^ = 0.19), with significantly higher peak knee abduction moments for bilateral landings compared to unilateral tasks.

Peak ankle dorsiflexion moments were significantly affected by the task (*p <* 0.001, ηp^2^ = 0.76) and significantly higher in males compared to females only for the two rotational tasks (*p =* 0.002, ηp^2^ = 0.10) (Figure 3). There was also a significant interaction effect between the task and sex (*p <* 0.001, ηp^2^ = 0.81) for the ankle dorsiflexion moment, with greater between-sex differences in the rotational compared to the straight landings. Peak ankle eversion moments differed among all four landing tasks (*p <* 0.001, ηp^2^ = 0.58), but not between sexes.

Peak vertical GRF, time to peak vertical GRF, knee angular velocity at IC, and knee stiffness were all significantly affected by the landing task (*p <* 0.001) (Table 1). Knee stiffness was higher for the three unilateral landings, compared to the bilateral task, and higher for the UL_CCW_ compared to ULs (*p <* 0.001). Peak vertical GRF was significantly higher in the two rotational landing tasks compared to both straight landings (*p <* 0.001). There was a significant main effect of sex on the knee stiffness, but no interaction between sex and the landing task. Males presented a significantly longer time to peak vertical GRF (*p <* 0.001) than females, but only during the clockwise rotational landing tasks. There were also significant interactions between sex and the task for peak vertical GRF and time to peak GRF (Table 1), with differences between males and females being larger in the rotational compared to the straight landing tasks.

**Table 1:** Sex and task effects for peak GRF, time to peak GRF, knee adduction velocity, and knee stiffness in all four landing tasks

	Sex	BL_S_	UL_S_	UL_CCW_	UL_CW_	*p*-value (η_p_^2^ )		
						Task effect	Sex effect	Interaction effect
Peak GRF (BW)	Male Female	2.62 ± 1.03 2.10 ± 0.69	3.47 ± 0.81 3.51 ± 0.68	4.12 ± 1.28 4.06 ± 0.83	3.90 ± 0.87 4.11 ± 0.85	<0.01 (0.37)	0.34 (0)	0.02 (0.02)
Time to peak GRF (s)	Male Female	0.05 ± 0.01 0.05 ± 0.01	0.06 ± 0.01 0.06 ± 0.01	0.06 ± 0.01 0.05 ± 0.01	0.06 ± 0.01 0.05 ± 0.01	0.013 (0.03)	0.38 (0)	<0.01 (0.04)
Knee adduction velocity at IC (deg/s)	Male Female	87 ± 64 66 ± 80	72 ± 48 63 ± 62	63 ± 59 64 ± 61	55 ± 65 40 ± 77	<0.01 (0.03)	0.06 (0.01)	0.57 (0.01)
Knee stiffness (Nm/deg)	Male Female	2.3 ± 0.8 1.7 ± 0.6	2.9 ± 1.4 2.2 ± 0.8	3.3 ± 1.3 2.6 ± 1.4	3 ± 1.1 2.3 ± 1.1	<0.01 (0.94)	<0.01 (0.88)	0.99 (0)

BL_s_ = straight bilateral landing, UL_s_ = straight unilateral landing, UL_CCW_ = counterclockwise unilateral landing, UL_CW_ = clockwise unilateral landing, GRF = ground reaction force, IC = initial contact, η_p_^2^ = effect size

## Discussion

This study explored the effects of rotational landings on lower-limb kinematics and kinetics, and the sex-specific responses to rotational compared to straight landings. It was hypothesised that rotational landings would emphasise kinematic and kinetic factors typically examined for ACL-injury screening purposes and could highlight between-sex differences in lower-limb biomechanics more adequately than straight landing tasks.

Two variables were found to be most sensitive to rotational landings, i.e., hip flexion angles were significantly higher at IC during UL_CW_ and UL_CCW_ compared to the straight unilateral landing task (Figure 2), and peak vertical GRF was greater during rotational landings compared to both straight landing tasks. These findings provide support for the first hypothesis and the notion that rotational landings can emphasise mechanical characteristics in the lower limbs and thus support the identification of individuals at an increased risk of ACL injuries (Fox et al., 2016; Schweizer et al., 2022). Nevertheless, the effects of introducing rotational (multiplanar) elements in the functional landing task were limited for other kinematic and kinetic characteristics. Rotational landings can thus be recommended as beneficial to be included for injury-risk screening practice, especially since both hip flexion at IC and peak vertical GRF are deemed to be of importance based on previous research (Kipp et al., 2011; Padua and DiStefano, 2009).

The ability to identify between-sex differences is essential to enhance understanding of the current ACL injury-rate disparities between males and females (Agel et al., 2005; Arendt and Dick, 1995; Waldén et al., 2011). Knee flexion angles at IC were higher and ankle dorsiflexion moments lower for female athletes in both rotational landing tasks, but not the straight landing tasks, with a significant task-sex interaction for the latter. Likewise, between-sex differences in the hip adduction angle (UL_CCW_), the knee abduction angle (UL_CCW_), and time to peak GRF (UL_CW_; significant interaction), and the ankle eversion moment (UL_CW_) were only identified during a rotational landing task, but not during straight landings. These results support our second hypothesis and indicate that rotational landings have the capacity to highlight lower-limb kinematic and kinetic differences between males and females, which cannot be identified during straight landings. However, it should be noted that this capacity applies to a limited number of biomechanical characteristics only, the number of which is further reduced when interaction effects are considered. Previous research has demonstrated that ankle moments (Tait et al., 2022) and peak GRF timing (Miranda et al., 2013), the two variables for which we observed a significant interaction, may be associated with either increased knee loading or previous ACL injury, suggesting rotational landings can help identify sex-specific differences in biomechanical characteristics related to ACL injury.

Besides an emphasis on several ACL-injury related and sex-specific characteristics, our results indicate distinct relevance of the unilateral nature of landings. For example, landing characteristics were found to be significantly higher (hip flexion and adduction moment, knee flexion moment and stiffness, ankle plantarflexion angle, and dorsiflexion and eversion moment) or lower (knee flexion angle and abduction moment) for all unilateral landing tasks compared to the bilateral task, but no or minimal differences were observed between straight and rotational unilateral landings. Similarly, all unilateral landing tasks (but not the bilateral task) revealed significant differences between males and females for the hip flexion angles at IC, with no between-unilateral differences found. Since ACL injuries are known to primarily occur during unilateral landings across a wide range of sports (Cochrane et al., 2007; Della Villa et al., 2020; Koga et al., 2010; Olsen et al., 2004), unilateral tasks have previously been suggested as key movements to be included in ACL-injury risk screening practice (Fox et al., 2016; Schweizer et al., 2022). The present study thus provides additional support for the use of unilateral rather than bilateral landing tasks, which can be further complemented by including rotational elements.

Several limitations of the presented work and avenues for further research need to be highlighted. First, this work employed a lower-limb Plug-in-Gait marker set, which has limited ability to accurately assess movement in the transverse plane. Future studies may seek to further examine the effects of rotational landings on, e.g., internal knee rotation using a full six-degree-of-freedom marker set. Second, to achieve a successful rotational landing, participants had to perform a minor hop of the box rather than a slightly more passive drop, as was possible during the straight landing tasks. Although participants were carefully instructed to reduce the required hop of the box and visually assessed during each trial, this discrepancy between tasks may have contributed to slight variations in movement strategies during the landing task execution. Third, only a limited number of rotational tasks were included in the present exploration, which can explain that not all kinematic and kinetic variables were (substantially) affected by the rotational component. Additional, and possibly more challenging, multiplanar rotational landing tasks (e.g., including larger rotations) or conditions (e.g., landing from greater heights) may help further emphasise common ACL-injury risk factors and between-sex differences during landings.

## Conclusions

This study explored distinct kinematic and kinetic adjustments to rotational landings and sex-specific responses, with the purpose of informing ACL-injury risk screening practice. Rotational landings emphasised specific biomechanical characteristics (hip flexion at IC and peak vertical GRF) and between-sex differences (peak ankle dorsiflexion and time to peak vertical GRF). Moreover, our results demonstrate and further reinforce the importance of implementing unilateral landings as functional tasks used for injury screening. Together, these outcomes provide additional support for the use of rotational, as well as unilateral elements, for ACL-injury risk screening.

## References

[ref1] Agel, J., Arendt, E. A., & Bershadsky, B. (2005). Anterior cruciate ligament injury in national collegiate athletic association basketball and soccer: a 13-year review. *American Journal of Sports Medicine*, 33(4), 524–531. 10.1177/0363546504269937 15722283

[ref2] Arendt, E., & Dick, R. (1995). Knee injury patterns among men and women in collegiate basketball and soccer: NCAA data and review of literature. *American Journal of Sports Medicine*, 23(6), 694–701. 10.1177/036354659502300611 8600737

[ref3] Bahr, R. (2016). Why screening tests to predict injury do not work—and probably never will…: a critical review. *British Journal of Sports Medicine*, 50(13), 776–780. 10.1136/bjsports-2016-096256 27095747

[ref4] Bates, N. A., Schilaty, N. D., Nagelli, C. V., Krych, A. J., & Hewett, T. E. (2019). Multiplanar loading of the knee and its influence on anterior cruciate ligament and medial collateral ligament strain during simulated landings and noncontact tears. *American Journal of Sports Medicine*, 47(8), 1844–1853. 10.1177/0363546519850165 31150273 PMC6988507

[ref5] Bathe, C., Fennen, L., Heering, T., Greif, A., & Dubbeldam, R. (2023). Training interventions to reduce the risk of injury to the lower extremity joints during landing movements in adult athletes: a systematic review and meta-analysis. *BMJ Open Sport & Exercise Medicine*, 9(2), e001508. 10.1136/bmjsem-2022-001508 PMC10254820 37304892

[ref6] Beaulieu, M. L., & McLean, S. G. (2012). Sex-dimorphic landing mechanics and their role within the noncontact ACL injury mechanism: evidence, limitations and directions. *Sports Medicine, Arthroscopy, Rehabilitation, Therapy & Technology*, 4, 1–13. 10.1186/1758-2555-4-10 PMC3320528 22420302

[ref7] Butler, R. J., Willson, J. D., Fowler, D., & Queen, R. M. (2013). Gender differences in landing mechanics vary depending on the type of landing. *Clinical Journal of Sport Medicine*, 23(1), 52–57. 10.1097/JSM.0b013e318259efa0 22678111

[ref8] Cochrane, J. L., Lloyd, D. G., Buttfield, A., Seward, H., & McGivern, J. (2007). Characteristics of anterior cruciate ligament injuries in Australian football. *Journal of Science and Medicine in Sport*, 10(2), 96–104. 10.1016/j.jsams.2006.05.015 16807104

[ref9] Cohen, J. (2013). Statistical power analysis for the behavioral sciences. Routledge.

[ref10] Della Villa, F., Buckthorpe, M., Grassi, A., Nabiuzzi, A., Tosarelli, F., Zaffagnini, S., & Della Villa, S. (2020). Systematic video analysis of ACL injuries in professional male football (soccer): Injury mechanisms, situational patterns and biomechanics study on 134 consecutive cases. *British Journal of Sports Medicine*, 54(23), 1423–1432. 10.1136/bjsports-2019-101247 32561515

[ref11] Fagenbaum, R., & Darling, W. G. (2003). Jump landing strategies in male and female college athletes and the implications of such strategies for anterior cruciate ligament injury. *American Journal of Sports Medicine*, 31(2), 233–240. 10.1177/03635465030310021301 12642258

[ref12] Fox, A. S., Bonacci, J., McLean, S. G., Spittle, M., & Saunders, N. (2016). A systematic evaluation of field-based screening methods for the assessment of anterior cruciate ligament (ACL) injury risk. *Sports Medicine*, 46, 715–735. 10.1007/s40279-015-0443-3 26626070

[ref13] Goerger, B. M., Marshall, S. W., Beutler, A. I., Blackburn, J. T., Wilckens, J. H., & Padua, D. A. (2015). Anterior cruciate ligament injury alters preinjury lower extremity biomechanics in the injured and uninjured leg: the JUMP-ACL study. *British Journal of Sports Medicine*, 49(3), 188–195. 10.1136/bjsports-2013-092982 24563391

[ref14] Hewett, T. E., Myer, G. D., Ford, K. R., Heidt, R. S., Jr, Colosimo, A. J., McLean, S. G., ... & Succop, P. (2005). Biomechanical measures of neuromuscular control and valgus loading of the knee predict anterior cruciate ligament injury risk in female athletes: a prospective study. *American Journal of Sports Medicine*, 33(4), 492–501. 10.1177/0363546504269591 15722287

[ref15] Jones, H. S. R., Verheul, J., Daniels, K. A. J., Stiles, V. H., & Moore, I. S. (2025). Differences in vertical and lower-limb joint stiffness in RTS assessments between ACLR patients and non-injured controls. *Journal of Sports Sciences*, 43(8), 738-745. 10.1080/02640414.2025.2474340 40051018

[ref16] Knudson, D. (2009). Significant and meaningful effects in sports biomechanics research. *Sports Biomechanics*, 8(1), 96–104. 10.1080/14763140802629966 19391497

[ref17] Koga, H., Nakamae, A., Shima, Y., Iwasa, J., Myklebust, G., Engebretsen, L., Bahr, R., & Krosshaug, T. (2010). Mechanisms for noncontact anterior cruciate ligament injuries: Knee joint kinematics in 10 injury situations from female team handball and basketball. *American Journal of Sports Medicine*, 38(11), 2218–2225. 10.1177/0363546510373570 20595545

[ref18] Krosshaug, T., Steffen, K., Kristianslund, E., Nilstad, A., Mok, K. M., Myklebust, G., Andersen, T. E., Holme, I., Engebretsen, L. & Bahr, R. (2016). The vertical drop jump is a poor screening test for ACL injuries in female elite soccer and handball players: a prospective cohort study of 710 athletes. *American Journal of Sports Medicine*, 44(4), 874–883. 10.1177/0363546515625048 26867936

[ref19] Kunugi, S., Koumura, T., Myotsuzono, R., Masunari, A., Yoshida, N., Miyakawa, S., & Mukai, N. (2020). Directions of single-leg landing affect multi-segment foot kinematics and dynamic postural stability in male collegiate soccer athletes. *Gait & Posture*, 80, 285–291. 10.1016/j.gaitpost.2020.06.007 32570194

[ref20] Lephart, S. M., Ferris, C. M., Riemann, B. L., Myers, J. B., & Fu, F. H. (2002). Gender differences in strength and lower extremity kinematics during landing. *Clinical Orthopaedics and Related Research*, 401, 162–169. 10.1097/00003086-200208000-00019 12151893

[ref21] Leppänen, M., Pasanen, K., Kujala, U. M., Vasankari, T., Kannus, P., Äyrämö, S., ... & Parkkari, J. (2017). Stiff landings are associated with increased ACL injury risk in young female basketball and floorball players. *American Journal of Sports Medicine*, 45(2), 386–393. 10.1177/0363546516665810 27637264

[ref22] Maloney, S. J., & Fletcher, I. M. (2021). Lower limb stiffness testing in athletic performance: a critical review. *Sports Biomechanics*, 20(1), 109–130. 10.1080/14763141.2018.1460395 29768094

[ref23] Markolf, K. L., Burchfield, D. M., Shapiro, M. M., Shepard, M. F., Finerman, G. A., & Slauterbeck, J. L. (1995). Combined knee loading states that generate high anterior cruciate ligament forces. *Journal of Orthopaedic Research*, 13(6), 930–935. 10.1002/jor.1100130618 8544031

[ref24] Miranda, D. L., Fadale, P. D., Hulstyn, M. J., Shalvoy, R. M., Machan, J. T. & Fleming, B. C. (2013). Knee biomechanics during a jump-cut maneuver: effects of gender & ACL surgery. *Medicine and Science in Sports and Exercise*, 45(5), 942–951. 23190595 10.1249/MSS.0b013e31827bf0e4 PMC3594620

[ref25] Moses, B., Orchard, J., & Orchard, J. (2012). Systematic review: Annual incidence of ACL injury and surgery in various populations. *Research in Sports Medicine*, 20(3–4), 157–179. 10.1080/15438627.2012.68063 22742074

[ref26] Olsen, O. E., Myklebust, G., Engebretsen, L., & Bahr, R. (2004). Injury mechanisms for anterior cruciate ligament injuries in team handball: a systematic video analysis. *American Journal of Sports Medicine*, 32(4), 1002–1012. 10.1177/0363546503261724 15150050

[ref27] Padua, D. A. & DiStefano, L. J. (2009). Sagittal plane knee biomechanics and vertical ground reaction forces are modified following ACL injury prevention programs: a systematic review. *Sports Health*, 1(2), 165–173. 23015868 10.1177/1941738108330971 PMC3445071

[ref28] Pedley, J. S., Lloyd, R. S., Read, P. J., Moore, I. S., De Ste Croix, M., Myer, G. D., & Oliver, J. L. (2020). Utility of kinetic and kinematic jumping and landing variables as predictors of injury risk: a systematic review. *Journal of Science in Sport and Exercise*, 2, 287–304. 10.1007/s42978-020-00090-1

[ref29] Peterson, T., & Green, R. (2023). Assessing risk parameters of ACL injury via human pose estimation. *Orthopaedic Proceedings*, 105(3), 97. 10.1302/1358-992x.2023.3.097

[ref30] Quatman, C. E., Quatman-Yates, C. C., & Hewett, T. E. (2010). A ‘plane’ explanation of anterior cruciate ligament injury mechanisms. *Sports Medicine*, 40(9), 729–746. 10.2165/11534950-000000000-00000 20726620

[ref31] Sadeqi, S., Norte, G. E., Murray, A., Erbulut, D. U., & Goel, V. K. (2023). Effect of whole body parameters on knee joint biomechanics: implications for ACL injury prevention during single-leg landings. *American Journal of Sports Medicine*, 51(8), 2098–2109. 10.1177/03635465231174899 37259968

[ref32] Schweizer, N., Strutzenberger, G., Franchi, M. V., Farshad, M., Scherr, J., & Spörri, J. (2022). Screening tests for assessing athletes at risk of acl injury or reinjury—a scoping review. *International Journal of Environmental Research and Public Health*, 19(5), 2864. 10.3390/ijerph19052864 35270563 PMC8910677

[ref33] Serpell, B. G., Ball, N. B., Scarvell, J. M., & Smith, P. N. (2012). A review of models of vertical, leg, and knee stiffness in adults for running, jumping or hopping tasks. *Journal of Sports Sciences*, 30(13), 1347–1363. 10.1080/02640414.2012.710755 22845059

[ref34] Sinsurin, K., Srisangboriboon, S., & Vachalathiti, R. (2017). Side-to-side differences in lower extremity biomechanics during multi-directional jump landing in volleyball athletes. *European Journal of Sport Science*, 17(6), 699–709. 10.1080/17461391.2017.1308560 28394742

[ref35] Sinsurin, K., Vachalathiti, R., Jalayondeja, W., & Limroongreungrat, W. (2013). Different sagittal angles and moments of lower extremity joints during single-leg jump landing among various directions in basketball and volleyball athletes. *Journal of Physical Therapy Science*, 25(9), 1109–1113. 10.1589/jpts.25.1109 24259925 PMC3818772

[ref36] Tait, D. B., Newman, P., Ball, N. B. & Spratford, W. (2022). What did the ankle say to the knee? Estimating knee dynamics during landing—A systematic review and meta-analysis. *Journal of Science and Medicine in Sport*, 25(2), 183–191. 10.1016/j.jsams.2021.08.007 34509342

[ref37] Taylor, J. B., Ford, K. R., Schmitz, R. J., Ross, S. E., Ackerman, T. A., & Shultz, S. J. (2017). Biomechanical differences of multidirectional jump landings among female basketball and soccer players. *Journal of Strength & Conditioning Research*, 31(11), 3034–3045. 10.1519/JSC.0000000000001785 29065078

[ref38] Waldén, M., Hägglund, M., Magnusson, H., & Ekstrans, J. (2011). Anterior cruciate ligament injury in elite football: a prospective three-cohort study. *Knee Surgery, Sports Traumatology, Arthroscopy*, 19(1), 11–19. 10.1007/s00167-010-1170-9 20532869

